# Thrombocytopenia in carriers and patients with antiphospholipid syndrome: insights from the nationwide START-APS registry

**DOI:** 10.1007/s11739-026-04416-9

**Published:** 2026-06-16

**Authors:** Francesco Del Sole, Tommaso Bucci, Danilo Menichelli, Emilia Antonucci, Daniela Poli, Vittorio Pengo, Paul R. J. Ames, Doris Barcellona, Piera Sivera, Attilia Maria Pizzini, Giuseppe Vercillo, Daniele Pastori, Pasquale Pignatelli, START-A. P. S. Investigators^, Danilo Menichelli, Daniela Poli, Doris Barcellona, Piera Sivera, Attilia Maria Pizzini, Daniele Pastori, Pasquale Pignatelli, Raffaella Benedetti, Davide Imberti, Silvia Bozzato, Giovanna Colombo, Laura Banov, Elisa Bison, Verusca Brusegan, Luca Barcella, Francesca Schieppati, Anna Falanga, Chiara Ambaglio, Sara Gamba, Marina Marchetti, Eugenio Bucherini, Antonio Chistolini, Maria Sophia Cotelli, Erica De Candia, Alice Lipari, Vittorio Fregoni, Paolo Gresele, Ariela Hoxha, Corrado Lodigiani, Alberto Maino, Gessica Marchesini, Giuseppe Carli, Alberto Tosetto, Vincenzo Oriana, Pancani Roberta, Simona Pedrini, Gian Marco Podda, Bianca Clerici, Elena Lotti, Domenico Prisco, Maria Canfora, Elena Silvestri, Luca Puccetti, Jacopo Agnelli Giacchello, Laura Contino, Roberto Santi, Luca Sarti, Anna Aiello, Carmine Spataro, Sophie Testa, Rossella Morandini, Oriana Paoletti, Andrea Toma, Antonella Tufano, Vercillo Giuseppe, Nello Zanatta, Beniamino Zalunardo, Adriana Visonà, Michela Frulla

**Affiliations:** 1 Department of Anatomical, Histological, Forensic Medicine and Orthopaedic Sciences, Sapienza University of Rome, Rome, Italy; 2 Internal, Vascular and Emergency Medicine–Stroke Unit, University of Perugia, Perugia, Italy; 3 Liverpool Centre for Cardiovascular Sciences, University of Liverpool, Liverpool John Moores University and Liverpool Heart and Chest Hospital, Liverpool, UK; 4 Department of General Surgery, Surgical Specialty and Anesthesiology, Sapienza University of Rome, Rome, Italy; 5 Arianna Anticoagulazione Foundation, Bologna, Italy; 6 Thrombosis Centre, Azienda Ospedaliero-Universitaria Careggi, Florence, Italy; 7 Cardio-Immunology and Thrombosis Research Laboratory, University of Padova, Padua, Italy; 8 Department of Haematology, Dumfries Royal Infirmary, Cargenbridge, Dumfries UK; 9 Immune Response & Vascular Disease, iNOVA, 4Health, Nova Medical School, Nova University Lisbon, Lisbon, Portugal; 10 Struttura Dipartimentale Di Emostasi E Trombosi, AOU Di Cagliari, Cagliari, Italy; 11 SCDU Ematologia, Ospedale Mauriziano Torino, Torin, Italy; 12 UOC Medicina A- Ospedale Maggiore, Bologna, Italy; 13 UO Patologia Clinica-IRCCS Regina Elena National Cancer Institute, Rome, Italy; 14 Department of Medical and Cardiovascular Sciences, University of Rome “Sapienza”, 00161 Rome, Italy; 15 IRCCS Neuromed, Località Camerelle, Pozzilli, Isernia Italy

**Keywords:** Antiphospholipid syndrome, Thrombocytopenia, Carriers, Antiphospholipid antibodies

## Abstract

Patients with thrombotic antiphospholipid syndrome (APS) or subjects with persistent presence of antiphospholipid antibodies (aPLA), defined “carriers”, may develop thrombocytopenia. However, the prevalence and clinical characteristics associated with thrombocytopenia remain poorly understood. We aimed to describe the prevalence and the clinical characteristics associated with thrombocytopenia in APS patients and aPLA carriers included in the Survey on AnTicoAgulated Patients-RegisTry on antiphospholipid antibodies (START-APS), a multicentre prospective registry. Thrombocytopenia was defined as a platelet count < 150 × 10^9^/L, while moderate-to-severe form was defined as < 100 × 10^9^/L. Logistic regression analysis was performed, and results were expressed as Odds Ratio (OR) and 95% confidence interval (95%CI). A total of 464 patients were included (148 aPLA carriers, 316 APS), mean age was 57.3 ± 15.6 years, and 66.5% were women. 63 (13.6%) patients presented with thrombocytopenia; 15.2% in APS patients and 10.1% in aPLA carriers (*p* = 0.138). Overall, triple positive pattern (OR 1.90, *p* = 0.002), livedo reticularis (OR 3.57, *p* = 0.029), heart failure (OR 4.52, *p* = 0.039), and female sex (OR 0.59, *p* = 0.011) were associated with thrombocytopenia. Triple positivity (OR 5.44, *p* < 0.001) and history of myocardial infarction (OR 6.52, *p* = 0.009) were associated with moderate-to-severe thrombocytopenia. Thrombocytopenia may occur early also in aPLA carriers, raising to 15% in APS patients, especially in those presenting with triple positivity and cardiovascular involvement. This association was confirmed also with moderate-to-severe thrombocytopenia.

**Supplementary Information:**

The online version contains supplementary material available at https://doi.org/10.1007/s11739-026-04416-9.

## Introduction

Thrombotic antiphospholipid syndrome (APS) is a systemic autoimmune condition characterized by venous, arterial, or microvascular events and the persistent presence of antiphospholipid antibodies (aPLA), such as anticardiolipin antibodies (aCL) and anti-β2 glycoprotein I antibodies (aβ2GPI) or Lupus Anticoagulant (LA) [1] [2–4]. APS can be primary or associated with other autoimmune conditions such as Systemic Lupus Erythematosus (SLE); in the latter case, it is defined as secondary APS and is associated with a higher frequency of clinical manifestations, including cardiac valvular involvement and thrombocytopenia [5].

Beyond overt APS, patients with persistently positive aPL or LA in the absence of clinical manifestations are defined as aPLA carriers. aPLA positivity has been reported in a wide range of clinical conditions, including autoimmune disease (e.g., SLE, systemic sclerosis, rheumatoid arthritis), malignancies, infectious diseases, and in otherwise healthy individuals[6]. While the impact on mortality remains uncertain and thrombotic risk in carriers is not clearly established, high-risk aPLA profiles may warrant consideration of primary antithrombotic prophylaxis, given their potential association with thrombotic risk [7].

Thrombocytopenia is a common finding in aPLA positive patients, with reported prevalences, ranging from 10 to 40% in different cohorts [8–11]. The 2023 ACR/EULAR APS classification criteria emphasize the overall clinical phenotype over isolated thrombotic or laboratory findings [4]. Evidence suggesting a worse prognosis in patients with thrombocytopenia [12–14] highlights the need to better characterize its prevalence and clinical features, with potential implications for identifying higher thrombotic risk profiles.

For these reasons, we aimed to evaluate the prevalence and clinical characteristics of thrombocytopenia in a nationwide Italian cohort of APS patients and aPLA carriers, with particular focus on factors associated with moderate-to-severe thrombocytopenia.

## Methods

The Survey on AnTicoAgulated Patients-RegisTry on antiphospholipid antibodies (START-APS) is a branch of the START2-Registry (NCT02219984). It is a multicentric prospective registry for data from Centres for the Diagnosis of Thrombosis and Surveillance of Antithrombotic Therapies (FCSA) promoted by Arianna Foundation on Anticoagulation in Italy.

Approval was obtained from the Institutional Review Board or Ethics Committee at each participating center. All the patients signed an informed consent at time of enrollment in the Registry, and the study was conducted according to the ethical principles for medical research as set out in the Declaration of Helsinki.

Data were collected through an electronic clinical report form (e-CRF) accessible to each participating center via secure credentials. Patients were consecutively enrolled from registry initiation (October 2011) to data extraction (May 2025). We performed a cross-sectional analysis of the prospective START-APS registry. We evaluated baseline data of all patients, collected during the first outpatient visit in each participating center.

### Study population and baseline features

Patients presenting with a first episode of venous or arterial thrombosis were included as APS patients in the study if tested positive for one or more aPLA criteria (aCL, aβ2GPI or LA), confirmed after 12 weeks. Patients were defined as carriers if tested positive for one or more aPLA criteria without clinical evidence of arterial/venous thrombotic event or obstetrical manifestations. The diagnosis of APS and the definition of aPLA carriers were defined according to Sapporo criteria [2, 3], since the registry was started before the updated definition of the ACR/EULAR 2023 classification criteria. The laboratory assessment and determination of aPLA criteria have been described in a previous study [15].

Demographic, laboratory, and clinical data were entered in the web-based case report form. Baseline clinical features included cardiovascular risk factors, chronic respiratory, liver or kidney diseases, presence of autoimmune disorders, and frailty risk factors and therapy. Anticoagulation treatment was administered according to clinical guidelines and physician judgment.

### Thrombocytopenia definition

All laboratory data were collected from patients’ blood samples’ exams results brought at an outpatients visit at baseline. Thrombocytopenia was defined from the baseline laboratory test as platelet count less than 150 × 10^9^/L[16, 17], while moderate-–severe thrombocytopenia was defined as platelet count less than 100 × 10^9^/L.

### Study objectives

The primary objective of our study was to estimate the prevalence of thrombocytopenia in APS patients and aPLA carriers. We described the clinical and laboratory characteristics of patients with and without thrombocytopenia and assessed clinical factors associated with thrombocytopenia.

As secondary objectives, we aimed to evaluate the clinical features associated with moderate-to-severe thrombocytopenia.

### Statistical analysis

Descriptive analysis was performed according to presence of thrombocytopenia and aPLA carrier/APS condition. Categorical variables are reported as number or percentage and Pearson’s Chi-square test was used to compare proportions. Continuous variables are expressed as mean ± standard deviation or median and interquartile range (IQR), when appropriate. The distribution of continuous variables was evaluated using the Kolmogorov–Smirnov test. Student’s *t* test was used to compare mean in normal distribution, while Mann–Whitney test was used to compare median values in variables with a non-parametric distribution.

A stepwise logistic regression analysis was used to calculate Odds Ratio (OR) and their 95% confidence intervals (95%CI) to evaluate clinical factors associated with thrombocytopenia and moderate-to-severe thrombocytopenia. Restricted cubic splines were performed, and Wald test was used to assess linearity.

Data analysis was conducted using SPSS (IBM SPSS-25, SPSS Inc.), with a two-tailed significance level of *α* = 0.05 for all tests.

## Results

### Clinical cohort characteristics and prevalence of thrombocytopenia in APS patients and aPLA carriers

A total of 464 patients (148 aPLA carriers, 316 APS) were included from the registry (Table 1). The mean age was 57.3 ± 15.6 years, and 308 patients were women (66.5%). A total of 63 patients was affected with thrombocytopenia. Overall, the prevalence of thrombocytopenia was 13.6% with no significant difference in prevalence between APS patients and aPLA carriers (15.2% vs 10.1%, *p* = 0.138).

**Table 1:** Clinical cohort characteristics according to thrombocytopenia

	Total (*N* = 464)	No thrombocytopenia (*N* = 401)	Thrombocytopenia (*N* = 63)	*p* value
**Clinical features**				
*Age (years)*	57.30 ± 15.64	57.29 ± 15.77	57.34 ± 15.00	0.982
*Women n (%)*	308 (66.5)	274 (68.3)	34 (54.0)	0.025
*BMI (mean)*	25.92 ± 4.98	25.99 ± 5.10	25.48 ± 4.21	0.460
*Cancer history n (%)*	39 (8.4)	33 (8.2)	6 (9.5)	0.731
*Diabetes mellitus n (%)*	27 (5.8)	22 (5.5)	5 (7.9)	0.440
*Hypertension n (%)*	165 (35.6)	140 (34.9)	25 (39.7)	0.462
*Cerebral vascular disease n (%)*	61 (13.1)	51 (12.7)	10 (15.9)	0.491
*Ischemic heart disease n (%)*	17 (3.7)	14 (3.5)	3 (4.8)	0.618
*Valvular heart disease n(%)*	17 (3.7)	13 (3.2)	4 (6.3)	0.220
*Heart failure n (%)*	4 (0.9)	2 (0.5)	2 (3.2)	0.033
*Peripheral artery disease n (%)*	20 (4.3)	20 (5.0)	0 (0.0)	0.070
*Chronic lung disease n (%)*	26 (5.6)	23 (5.7)	3 (4.8)	0.755
*Liver cirrhosis n (%)*	3 (0.6)	2 (0.5)	1 (1.6)	0.316
*Chronic kidney disease n (%)*	18 (3.9)	14 (3.5)	4 (6.3)	0.275
*Hypothyroidism n (%)*	57 (12.3)	50 (12.5)	7 (11.1)	0.760
*Hyperthyroidism n (%)*	5 (1.1)	3 (0.7)	2 (3.2)	0.083
*Anemia n (%)*	25 (5.4)	20 (5.0)	5 (7.9)	0.335
*Hemolytic anaemia n (%)*	12 (2.6)	7 (1.7)	5 (7.9)	0.004
*Smoking habit n (%)*	102 (22.0)	90 (22.4)	12 (19.0)	0.545
*Livedo reticularis n (%)*	7 (1.5)	3 (0.7)	4 (6.3)	0.001
*Carriers (%)*	148 (31.9)	133 (33.2)	15 (23.8)	0.138
**Frailty risk elements**				
*Cognitive impairment n (%)*	17 (3.7)	10 (2.5)	7 (11.1)	0.001
*Bed rest syndrome n (%)*	5 (1.1)	4 (1.0)	1 (1.6)	0.673
*Falling predisposition n (%)*	2 (0.4)	1 (0.2)	1 (1.6)	0.132
*Alcohol consumption n (%)*	17 (3.7)	14 (3.5)	3 (4.8)	0.618
Previous thrombotic event				
*Deep vein thrombosis n (%)*	183 (39.4)	152 (37.9)	31 (49.2)	0.088
*Pulmonary embolism n (%)*	63 (13.6)	50 (12.5)	13 (20.6)	0.079
*Superficial vein thrombosis n(%)*	45 (9.7)	42 (10.5)	3 (4.8)	0.154
Laboratory indexes				
*Heamoglobin (g/dl)*	13.32 ± 1.61	13.36 ± 1.57	13.09 ± 1.89	0.198
*Leucocytes (cells/mm*^*3*^*)*	6.55 ± 2.34	6.64 ± 2.40	6.06 ± 1.98	0.112
*Creatinine (mg/dl)*	0.89 ± 0.29	0.86 ± 0.24	1.01 ± 0.49	< 0.001
*AST (U/L)*	22.0 [17.0–29.0]	22.0 [17.0–28.0]	26.0 [17.0–31.0]	0.289
*ALT (U/L)*	22.0 [17.0–30.1]	22.0 [17.0–30.0]	23.0 [15.0–31.0]	0.874
*Triple positivity pattern n (%)*	113 (24.4)	86 (21.4)	27 (42.9)	< 0.001
**Therapy**				
*Antiplatelet n (%)*	171 (36.9)	152 (37.9)	19 (30.2)	0.236
*Anticoagulant n (%)*	242 (52.2)	206 (51.4)	36 (57.1)	0.394
*Antiarrhythmic drugs n (%)*	20 (4.3)	16 (4)	4 (6.3)	0.391
*Lipid lowering treatment n (%)*	87 (18.8)	77 (19.2)	10 (15.9)	0.529
*ACE inhibitors n (%)*	51 (11.0)	40 (10.0)	11 (17.5)	0.077
*Calcium antagonists n (%)*	39 (8.4)	35 (8.7)	4 (6.3)	0.527
*Diuretics n (%)*	35 (7.5)	28 (7.0)	7 (11.1)	0.249
*ARB n (%)*	43 (9.3)	38 (9.5)	5 (7.9)	0.695
*Digoxin n (%)*	1 (0.2)	1 (0.2)	0	0.692
*PPI n (%)*	101 (21.8)	85 (21.2)	16 (25.4)	0.453
*Immunosuppressors n (%)*	55 (11.9)	44 (11.0)	11 (17.59)	0.139
*Corticosteroids n (%)*	52 (11.2)	39 (9.7)	13 (20.6)	0.011

*ACE angiotensin converting enzyme, ALT alanine aminotransferase, ARB angiotensin receptor blockers, AST aspartate transaminase, BMI body mass index, PPI proton pump inhibitor*

Thrombocytopenia was more frequently observed in men (68.3% vs 54%, *p* = 0.002). History of heart failure, hemolytic anemia, livedo reticularis, and cognitive impairment were significatively more common in patients with thrombocytopenia compared to patients without (Table 1). In addition, patients with thrombocytopenia showed significatively higher levels of serum creatinine (1.01 ± 0.49 vs 0.86 ± 0.24 mg/dl, *p* < 0.001), had more frequently a triple positivity pattern confirmed in two assays determination (42.9% vs 21.4%, *p* < 0.001), and were more frequently on corticosteroid treatment (20.6% vs 9.7%, *p* = 0.011) (Table 1). No further differences were observed between two groups on clinical characteristics, laboratory assessment, and therapy.

**Fig. 1:**
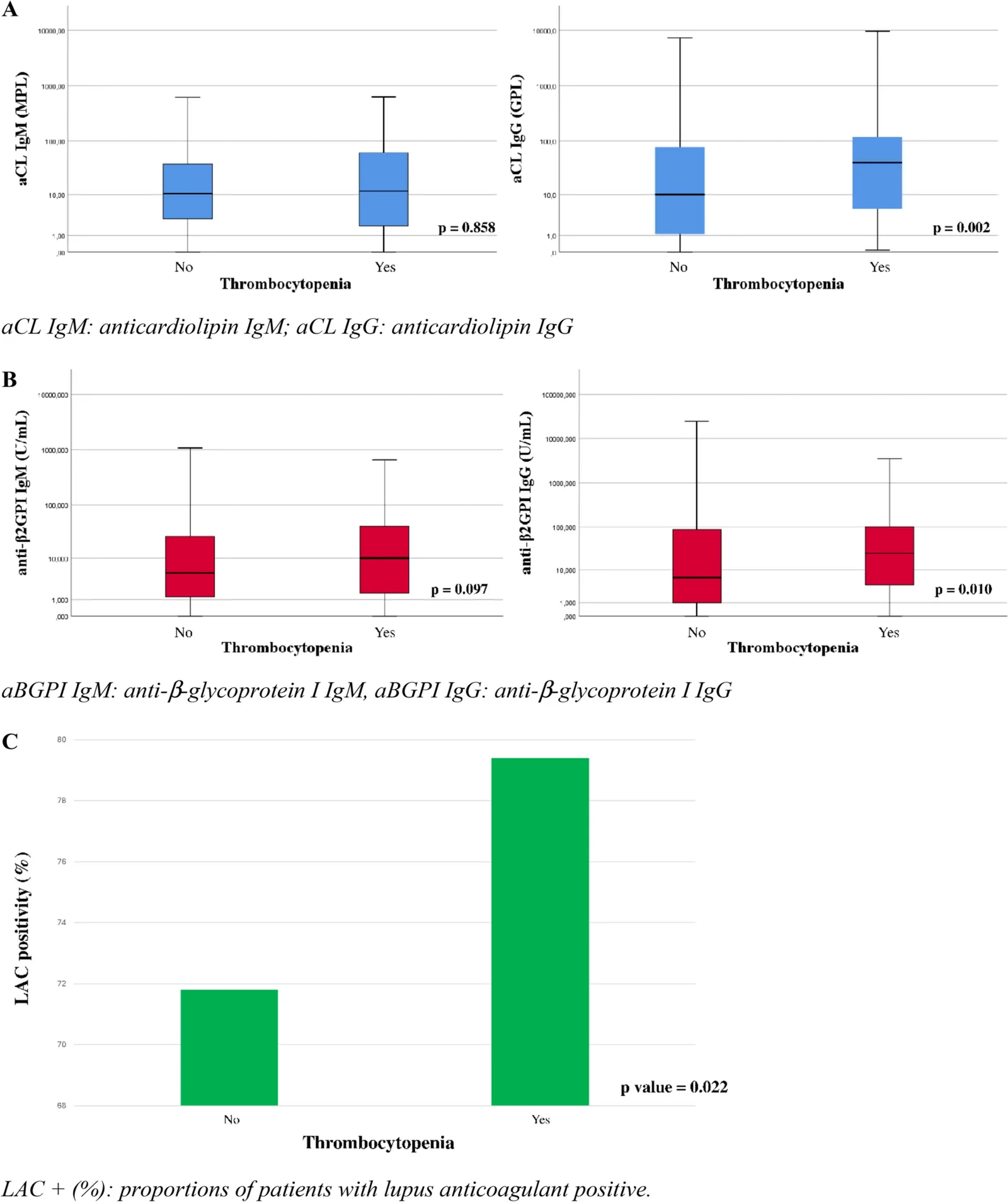
First antiphospholipid antibodies determination and concomitant thrombocytopenia. Panel A: anticardiolipin IgM and IgG, panel B: anti *β*-glycoprotein I IgM and IgG, and panel C: lupus anticoagulants positivity. *aCL IgM: anticardiolipin IgM; aCL IgG: anticardiolipin IgG*. *aBGPI IgM: anti-β-glycoprotein I IgM, aBGPI IgG: anti-β-glycoprotein I IgG*. *LAC* + *(%): proportions of patients with lupus anticoagulant positive*

**Fig. 2:**
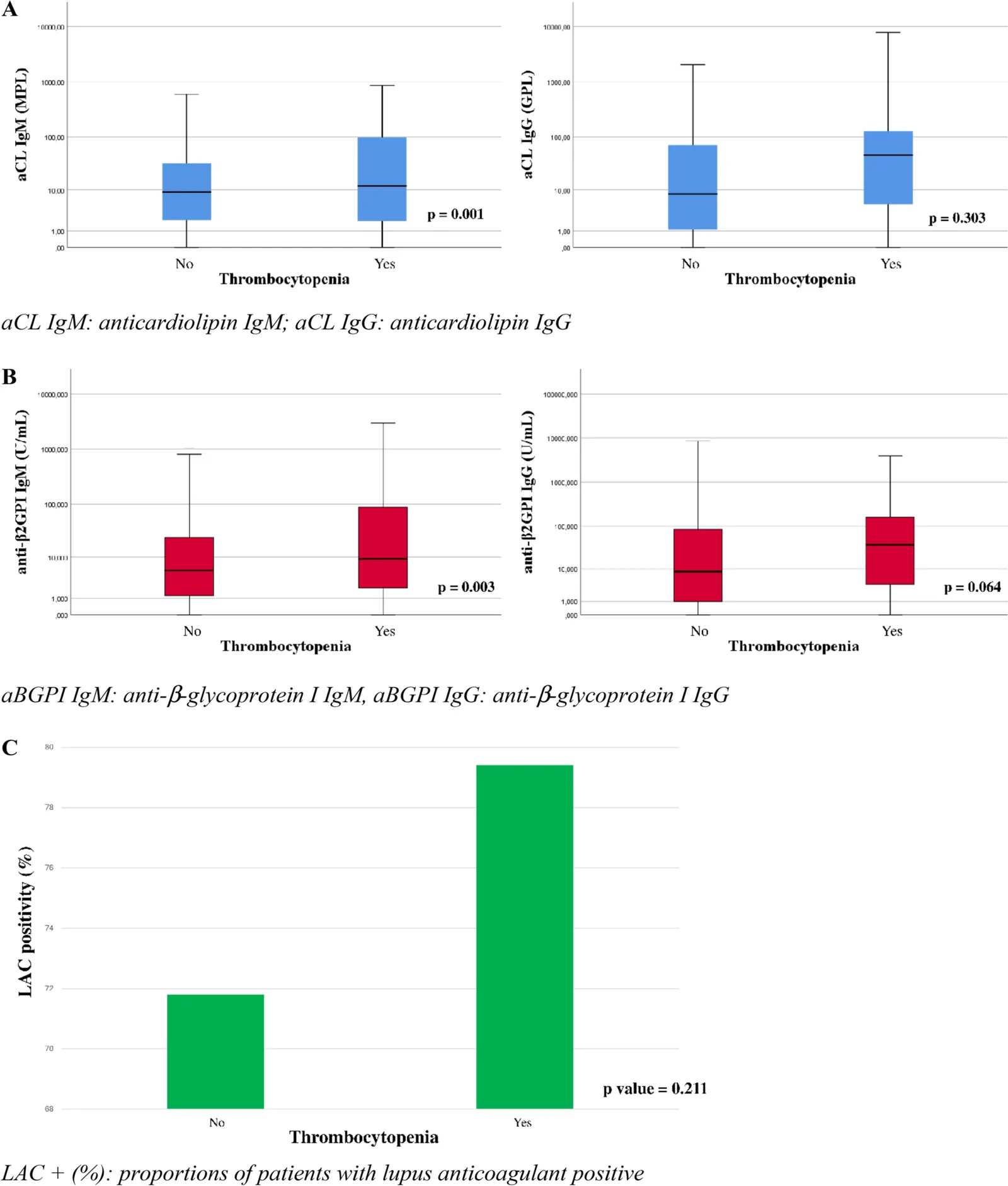
Second antiphospholipid antibodies determination and concomitant thrombocytopenia. Panel A: anticardiolipin IgM and IgG, panel B: anti *β*-glycoprotein I IgM and IgG, and panel C: lupus anticoagulants positivity. *aCL IgM: anticardiolipin IgM; aCL IgG: anticardiolipin IgG. aBGPI IgM: anti-β-glycoprotein I IgM, aBGPI IgG: anti-β-glycoprotein I IgG. LAC* + *(%): proportions of patients with lupus anticoagulant positive*

Patients affected with thrombocytopenia had higher levels of aCL IgG (40.0, IQR 5.0–120.0 vs 10.0, IQR 1.0–79.4, *p* = 0.002), aβ2GPI IgG (25.0, IQR 2.6–105.0 vs 6.4, IQR 1.0–88.3, *p* = 0.010) and had more frequently positive LAC essay at first determination (81.0% vs 66.6%, *p* = 0.022), while no statistically significant difference was observed in aCL IgM and aβ2GPI titers (Fig. 1, Panel A-C). In second determination, thrombocytopenic patients had higher aCL IgM titers (12.0, IQR 2.0–99.0 vs 9.1, IQR 2.1–34.0, *p* = 0.001) and aβ2GPI IgM (9.3, IQR 2.0–88.0 vs 5.3, IQR 1.2–24.2, *p* = 0.003), while no difference was observed in the LAC positivity and aCL IgG and aβ2GPI IgG titers (Fig. 2, Panel A-C). Triple positivity pattern confirmed in both determinations was more frequent in patients with thrombocytopenia (42.9% vs 21.4%, *p* < 0.001, Table 1).

Clinical characteristics according to aPLA carriers/APS status are shown in Table 2. aPLA carriers were more frequently women, treated with antiplatelets, and less frequently treated with lipid lowering therapy, proton pump inhibitors, and corticosteroids (Table 2). Carriers had higher levels of aCL IgG and aβ2GPI IgM compared to APS patients, but no other significant differences were observed between aPLA carriers and APS patients on aPLA and LA (Supplementary Figs. 1 and 2, Panel A-C). Among aPLA carriers, 15 had thrombocytopenia, while 48 had thrombocytopenia in the APS group. All clinical characteristics of patients with and without thrombocytopenia according to aPLA carriers/APS are shown in Supplementary Table 1.

**Table 2:** Clinical characteristics according to aPLA carriers/APS status

	APS (*N* = 316)	aPLA carriers (*N* = 148)	*p* value
**Clinical features**			
*Age (years)*	55.2 ± 16.2	53.8 ± 15.5	0.381
*Women n (%)*	195 (61.7)	113 (76.4)	0.002
*BMI mean*	26.0 ± 4.8	25.8 ± 5.3	0.663
*Cancer history n (%)*	21 (6.6)	18 (12.2)	0.046
*Diabetes mellitus n (%)*	17 (5.4)	10 (6.8)	0.555
*Hypertension n (%)*	118 (37.3)	47 (31.8)	0.241
*Heart failure (%)*	4 (1.3)	0 (0.0)	0.169
*Peripheral artery disease n (%)*	17 (5.4)	3 (2.0)	0.097
*Chronic lung disease n (%)*	22 (7.0)	4 (2.7)	0.063
*Chronic kidney disease n (%)*	14 (4.4)	4 (2.7)	0.369
*Hypothyroidism n (%)*	37 (11.7)	20 (13.5)	0.581
*Hyperthyroidism n (%)*	3 (0.9)	2 (1.4)	0.696
*Anemia n (%)*	21 (6.6)	4 (2.7)	0.080
*Hemolytic anemia n (%)*	9 (2.8)	3 (2.0)	0.604
*Thrombocytopenia (%)*	48 (15.2)	15 (10.1)	0.138
*Smoking habit n (%)*	66 (20.9)	36 (24.3)	0.405
*Livedo reticularis n (%)*	7 (2.2)	0 (0.0)	0.068
**Frailty risk elements**			
*Cognitive impairment n (%)*	14 (4.4)	3 (2.0)	0.199
*Bed rest syndrome n (%)*	5 (1.6)	0 (0.0)	0.124
*Falling predisposition n (%)*	1 (0.3)	1 (0.7)	0.582
*Former major bleeding n (%)*	8 (2.5)	1 (0.7)	0.177
*Alcohol consumption n (%)*	11 (3.5)	6 (4.1)	0.759
**Laboratory indexes**			
*Hemoglobin (g/dl)*	13.3 ± 1.7	13.4 ± 1.4	0.512
*Leukocytes (cells/mm*^*3*^*)*	6.7 ± 2.6	6.3 ± 1.9	0.176
*Creatinine (mg/dl)*	0.9 ± 0.3	0.9 ± 0.2	0.057
*AST (U/L)*	22.0 [17.0–29.0]	23.0 [17.0–30.5	0.445
*ALT (U/L)*	23.0 [16.5 − 30.0]	22.0 [17.0 − 31.0]	0.832
*Triple positivity pattern n (%)*	74 (23.6)	39 (26.4)	0.493
**Therapy**			
*Antiplatelet n (%)*	100 (31.6)	71 (48.0)	0.001
*Anticoagulant n (%)*	227 (71.8)	15 (10.1)	< 0.001
*Antiarrhythmic drugs n (%)*	15 (4.7)	5 (3.4	0.499
*Lipid lowering treatment n (%)*	71 (22.5)	16 (10.8)	0.003
*ACE inhibitors n (%)*	38 (12.0)	13 (8.8)	0.298
*Calcium antagonists n (%)*	31 (9.8)	8 (5.4)	0.111
*Diuretics n (%)*	29 (9.2)	6 (4.1)	0.051
*ARB n (%)*	28 (8.9)	15 (10.1)	0.659
*Digoxin n (%)*	1 (0.3)	0 (0.0)	0.493
*PPI n (%)*	83 (26.3)	18 (12.2)	0.001
*Immunosuppressors n (%)*	43 (13.6)	12 (8.1)	0.088
*Corticosteroids n (%)*	42 (13.3)	10 (6.8)	0.038

*ACE angiotensin converting enzyme, ALT alanine aminotransferase, aPLA antiphospholipid antibodies, APS antiphospholipid syndrome, ARB angiotensin receptor blockers, AST aspartate transaminase, BMI body mass index, PPI proton pump inhibitor*

**Fig. 3:**
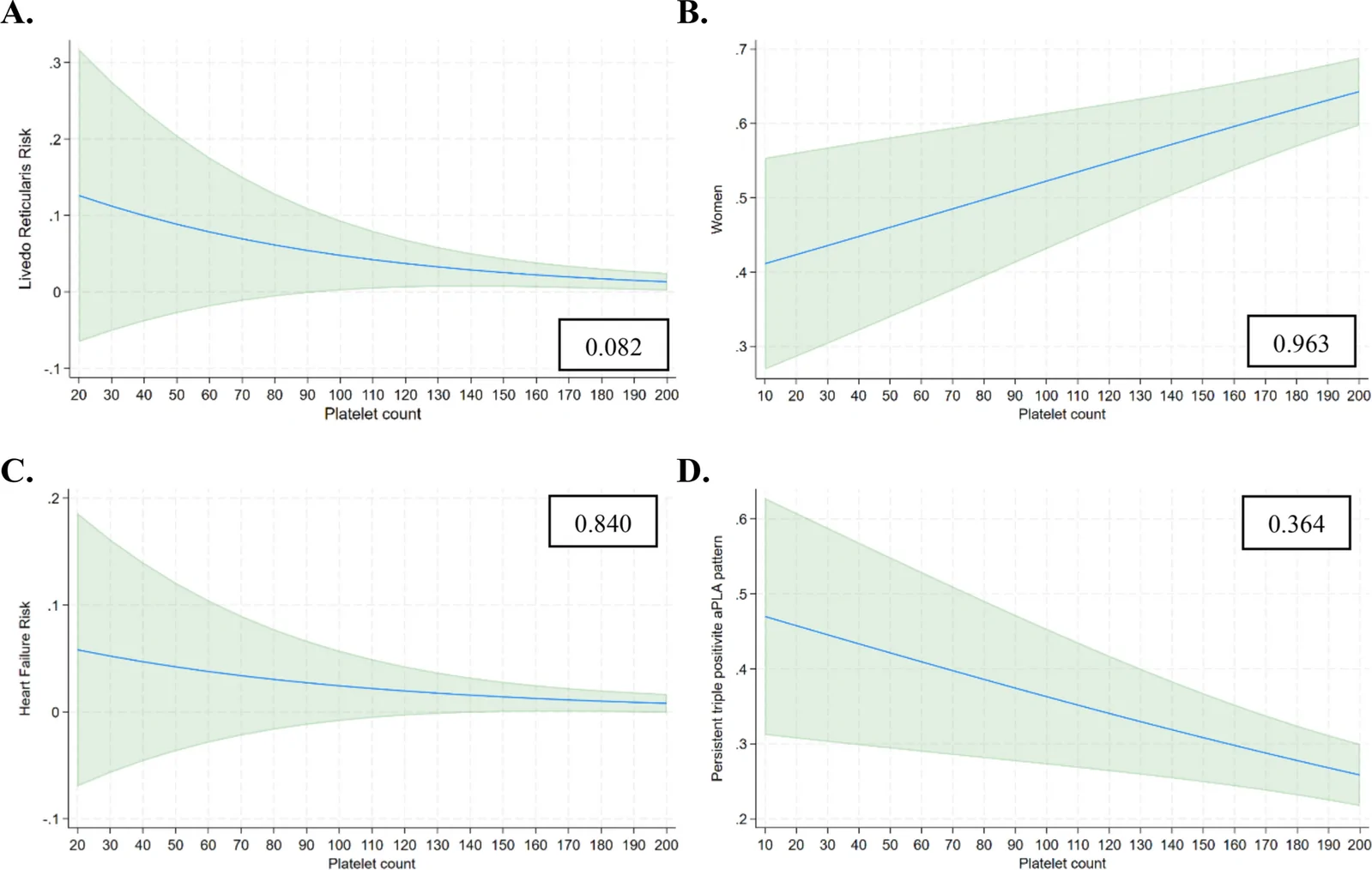
Restricted cubic spline evaluating the risk of: A. Livedo Reticularis, B. female sex, C. heart failure, and D. persistence of triple positive antibodies (aPLA) pattern according to platelet count

Thrombocytopenia has been directly associated with livedo reticularis (OR 3.57, 95%CI 1.14–11.25, *p* = 0.029) and heart failure (OR 4.52, 95%CI 1.08–18.98, *p* = 0.039) and inversely associated with female sex (OR 0.59, 95%CI 0.40—0.88 *p* = 0.011) at multivariable stepwise logistic regression (Table 3). A triple positivity aPLA pattern has been also associated with thrombocytopenia (OR 1.90, 95%CI 1.26–2.86, p 0.002). Restricted Cubic Spline confirmed these linear associations as shown in Fig. 3.

**Table 3:** Multivariable stepwise logistic regression of clinical factors associated with thrombocytopenia

	Odds ratio (95% confidence interval)	*p* value
Women	0.59 (0.40–0.88)	0.011
Livedo reticularis	3.57 (1.14–11.25)	0.029
Heart failure	4.52 (1.08–18.98)	0.039
Triple positivity aPLA pattern	1.90 (1.26–2.86)	0.002

*aPLA antiphospholipid antibodies*

### Clinical characteristics of moderate-to-severe thrombocytopenia

To evaluate clinical characteristics of patients with thrombocytopenia, we performed a subgroups analysis (Supplementary Table 2) according to thrombocytopenia degree (mild vs moderate-to-severe). We found no statistically significative difference between two groups apart from a higher prevalence of ischemic heart disease (11.1% vs 0.0%, *p* = 0.040) and a higher rate of corticosteroid treatment (33.3% vs 11.1%, *p* = 0.031) in the moderate-to-severe group.

To evaluate clinical factors associated with moderate-to-severe thrombocytopenia, we performed a multivariable stepwise logistic regression analysis (Supplementary Table 3) that showed a significant and direct association between triple positivity aPLA pattern (OR 5.44, 95%CI 2.27–13.04, *p* < 0.001), myocardial infarction (OR 6.52, 95%CI 1.59–26.77, *p* = 0.009), and moderate-to-severe thrombocytopenia.

## Discussion

In our study, thrombocytopenia was observed in 13.6% of patients, with a higher prevalence in APS compared to aPLA carriers, although this difference did not reach statistical significance. Patients with thrombocytopenia showed higher aCL IgG levels and a greater prevalence of LA and triple positivity. Livedo reticularis and heart failure were associated with thrombocytopenia, whereas female sex appeared to be protective.

We observed a lower prevalence compared to other studies. In a cohort of 1000 APS patients, thrombocytopenia was reported in 20–30% of cases [10]. This higher prevalence may be explained by the greater proportion of patients with secondary APS included in that study [10]. Consistently, Krause et al. [18], in a cohort 307 APS patients, reported a higher prevalence of thrombocytopenia in secondary APS compared to primary APS.

A further study by Galarza et al., including 211 APS patients, reported a thrombocytopenia prevalence of approximately 20%, slightly higher than in our cohort [19]. The cohort included a higher proportion of secondary APS, a younger population, and fewer women.

However, these studies did not report treatment data or measures of association, limiting the ability to identify clinical factors associated with thrombocytopenia and to draw further comparisons.

We observed a higher use of glucocorticoids in patients with thrombocytopenia. This finding is consistent with a previous study including 218 APS patients [12] and may reflect the role of glucocorticoids in the management of thrombocytopenia in APS, as supported by a meta-analysis of eight randomized clinical trials [14].

Finally, cognitive impairment was more frequent in patients with thrombocytopenia. This may be related to increased platelet activation, promoting microangiopathy, silent brain infarctions, and thromboembolism, with potential implications for cognitive decline [20], together with reduced circulating platelet levels.

We found a significant association between thrombocytopenia and both triple aPLA positivity and livedo reticularis. Notably, the association with triple positivity appeared to persist in patients with moderate-to-severe thrombocytopenia. These findings suggest that thrombocytopenia may identify a subset of patients with a more pronounced clinical phenotype, supporting its potential role as a marker of disease severity.

Patients with thrombocytopenia also showed a higher prevalence of heart failure, while moderate-to-severe thrombocytopenia was associated with coronary heart disease. Although the association between immune thrombocytopenia and coronary artery disease has been previously reported [21], evidence specifically addressing more severe forms of thrombocytopenia remains limited.

The role of thrombocytopenia APS-related vascular manifestations is complex. Immune-mediated platelet destruction may promote the release of prothrombotic microparticles[22] and increase the proportion of immature platelets, both contributing to a prothrombotic state [23]. On the other hand, autoantibody-mediated immune activation may induce endothelial damage through oxidative stress pathways [24].

Several mechanisms underline thrombocytopenia in APS patients and aPLA carriers [25]. aPLA activate endothelial cells, monocytes and platelets, promoting cytokine release, adhesion molecule expression, and platelet aggregation. They also upregulate tissue factor expression, enhancing activation of the coagulation cascade and thrombosis [26]. In addition to this prothrombotic milieu, immune-mediated platelet activation and increased platelet destruction further contribute to the development of thrombocytopenia [25, 27].

### Strengths and clinical implications

Our study had several strengths. First, the prospective design of the multicenter nationwide cohort registry permitted a real-world analysis of APS patients and aPLA carriers. In addition, we evaluated several clinical characteristics, including cardiovascular and non-cardiovascular comorbidities, frailty elements, treatment, and antibodies profiles permitting a well characterization of APS and aPLA cohorts. Furthermore, the use of multivariable logistic regression, unlike previous studies, led to identify and estimate the association between clinical features and thrombocytopenia. Besides, the evaluation of thrombocytopenia also in aPLA carriers represents a novelty of our study.

Finally, the characterization of thrombocytopenia and the clinical factors associated had clinical implications: indeed, as suggested by small previous studies [9, 28], thrombocytopenia may be associated with both thrombotic and bleeding events, and, as enhanced by our findings, with high autoimmune status that could influence the prognosis of these patients.

### Limitations

Our study had some limitations. First, we included in the analysis both aPLA carriers and APS cohort. Although we analyzed also separately the cohorts enrolled and we could estimate the prevalence of thrombocytopenia and clinical factors associated with it in both groups, the cohort may not result heterogeneous. Besides, the relatively small sample size and proportion of patients with thrombocytopenia leads to a cautious interpretation of the results.

In addition, the cross-sectional design of our study could not estimate the risk of bleeding and thrombotic events. Furthermore, our study has been conducted on a nationwide Italian cohort, and mainly, Caucasian patients were enrolled in the study. A further limitation of our studies consisted in the low number of patients with moderate-to-severe thrombocytopenia, that limit the robustness of our results in this subgroup of patients.

Another limitation in our study is due to the update in the APS classification from ACR/EULAR 2023 criteria, while the patients were partly enrolled in the registry before the clinical and laboratory criteria were updated. The impact of the revised criteria on the registry was already evaluated [15].

A further limitation of our study was the lack of data on anti-nuclear antibodies (ANA), which may be useful to assess autoimmune status, especially in patients with secondary APS.

## Conclusions

Thrombocytopenia is a frequent feature in APS and aPLA carriers, associated with both immunological manifestations and cardiovascular disease. Further studies are needed to better define its impact on thrombotic and hemorrhagic events and mortality.

## Supplementary Information

Below is the link to the electronic supplementary material.

Supplementary file1 (DOCX 451 KB)

## Data Availability

The data that support the findings of this study are available from the corresponding author upon reasonable request.
