# Supplementary material for: Thrombocytopenia in carriers and patients with antiphospholipid syndrome: insights from the nationwide START-APS registry
Source: Intern Emerg Med. 2026 Jun 16;21(7):2489–99. doi: 10.1007/s11739-026-04416-9 (PMC13694378; doi:10.1007/s11739-026-04416-9)
Supplement: Supplementary file 1 [file 11739_2026_4416_MOESM1_ESM.docx]

**Thrombocytopenia in carriers and patients with antiphospholipid syndrome: insights from the nationwide START-APS registry.**

*Supplementary material*

**Supplementary Table 1. Clinical characteristics according to antiphospholipid syndrome/carriers of antiphospholipid antibodies.**

**Supplementary Table 2. Clinical characteristics according to thrombocytopenia degree.**

**Supplementary Table 3. Multivariable stepwise logistic regression of clinical factors associated with moderate-to severe thrombocytopenia.**

**Supplementary Figure 1. Antiphospholipid antibodies in antiphospholipid syndrome patients and carriers. Panel A: anticardiolipin IgM and IgG, Panel B: anti β-glycoprotein I IgM and IgG, Panel C: lupus anticoagulants positivity.**

**Supplementary Figure 2. Antiphospholipid antibodies in antiphospholipid syndrome patients and carriers, second determination. Panel A: anticardiolipin IgM and IgG, Panel B: anti β-glycoprotein I IgM and IgG, Panel C: lupus anticoagulants positivity.**

**Supplementary Table 1. Clinical characteristics according to antiphospholipid syndrome/carriers of antiphospholipid antibodies.**

|  | **Carriers** | | | **Antiphospholipid Syndrome** | | |
| --- | --- | --- | --- | --- | --- | --- |
|  | **No thrombocytopenia (N = 133)** | **Thrombocytopenia (N = 15)** | **p-value** | **No thrombocytopenia (N = 268)** | **Thrombocytopenia (N = 48)** | **p-value** |
| *Age (years)* | 55.58 ± 15.77 | 58.60 ± 12.80 | 0.476 | 58.14 ± 15.72 | 56.94 ± 15.77 | 0.628 |
| *Women n (%)* | 104 (78.2) | 9 (60) | 0.195 | 170 (63.4) | 25 (52.1) | 0.093 |
| *BMI mean* | 25.83 ± 5.46 | 25.24 ± 3.68 | 0.688 | 26.07 ± 4.92 | 25.56 ± 4.40 | 0.504 |
| *Cancer history n (%)* | 15 (11.3) | 3 (20) | 0.267 | 18 (6.7) | 3 (6.3) | 0.601 |
| *Diabetes mellitus n (%)* | 9 (6.8) | 1 (6.7) | 0.732 | 13 (4.9) | 4 (8.3) | 0.248 |
| *Hypertension n (%)* | 41 (30.8) | 6 (40) | 0.325 | 99 (36.9) | 19 (39.6) | 0.422 |
| *Cerebral Vascular Disease n (%)* | NA | NA | - | 50 (18.7) | 10 (20.8) | 0.428 |
| *Ischemic Heart Disease n (%)* | NA | NA | - | 13 (4.9) | 3 (6.3) | 0.449 |
| *Heart Failure (%)* | 0 (0.0) | 0 (0.0) | - | 2 (0.7) | 2 (4.2) | 0.111 |
| *Peripheral Artery Disease n (%)* | 3 (2.3) | 0 (0.0) | 0.724 | 17 (6.3) | 0 (0.0) | 0.056 |
| *Chronic Lung Disease n (%)* | 4 (3.0) | 0 (0.0) | 0.649 | 19 (7.1) | 3 (6.4) | 0.564 |
| *Liver Cirrhosis n (%)* | 0 (0.0) | 1 (6.7) | 0.101 | 2 (0.7) | 0 (0.0) | 0.719 |
| *Chronic Kidney Disease n (%)* | 3 (2.3) | 1 (6.7) | 0.351 | 11 (4.1) | 3 (6.3) | 0.360 |
| *Hypothyroidism n (%)* | 18 (13.5) | 2 (13.3) | 0.671 | 32 (11.9) | 5 (10.4) | 0.494 |
| *Hyperthyroidism n (%)* | 1 (0.8) | 1 (6.7) | 0.193 | 2 (0.7) | 1 (2.1) | 0.391 |
| *Anaemia n (%)* | 3 (2.3) | 1 (6.7) | 0.351 | 17 (6.3) | 4 (8.3) | 0.399 |
| *Haemolytic Anaemia n (%)* | 2 (1.5) | 1 (6.7) | 0.276 | 5 (1.9) | 4 (8.3) | 0.033 |
| *Smoking Habit n (%)* | 34 (25.6) | 2 (13.3) | 0.241 | 56 (20.9) | 10 (20.8) | 0.582 |
| *Livedo reticularis n (%)* | 0 (0.0) | 0 (0.0) |  | 3 (1.1) | 4 (8.3) | 0.012 |
| **Frailty risk elements** | | | | | | |
| *Cognitive impairment n (%)* | 2 (1.5) | 1 (6.7) | 0.276 | 8 (3.0) | 6 (12.5) | 0.010 |
| *Bed rest syndrome n (%)* | 0 (0.0) | 0 (0.0) | - | 4 (1.5) | 1 (2.1) | 0.564 |
| *Falling predisposition n (%)* | 1 (0.8) | 0 (0.0) | 0.899 | 0 (0.0) | 1 (2.1) | 0.152 |
| *Former major bleeding n (%)* | 1 (0.8) | 0 (0.0) | 0.899 | 6 (2.2) | 2 (4.2) | 0.349 |
| *Alcohol consumption n (%)* | 5 (3.8) | 1 (6.7) | 0.479 | 9 (3.4) | 2 (4.2) | 0.518 |
| **Laboratory indexes** | | | | | | |
| *Haemoglobin (g/dl)* | 13.44 ± 1.36 | 13.06 ± 1.75 | 0.318 | 13.33 ± 1.66 | 13.09 ± 1.94 | 0.377 |
| *Leukocytes (cells/mm^3^)* | 6.29 ± 1.85 | 6.64 ± 1.93 | 0.555 | 6.86 ± 2.66 | 5.91 ± 1.99 | 0.037 |
| *Creatinine (mg/dl)* | 0.83 ± 0.18 | 0.99 ± 0.35 | 0.007 | 0.88 ± 0.27 | 1.02 ± 0.52 | 0.004 |
| *AST (U/L)* | 23.0 [18.3-27.0] | 26.0 [13.5-34.0] | 0.765 | 22.0 [17.0-28.0] | 26.0 [18.0-31.0] | 0.164 |
| *ALT (U/L)* | 22.0 [18.5-30.0] | 24.0 [13.3-37.3] | 0.723 | 22.0 [16.3-30.0] | 23.0 [16.0-29.5] | 0.982 |
| *Triple Positivity Pattern* *n (%)* | 29 (21.8) | 10 (66.7) | <0.001 | 57 (21.3) | 17 (35.4) | 0.033 |
| **Therapy** | | | | | | |
| *Antiplatelet n (%)* | 65 (48.9) | 6 (40) | 0.354 | 87 (32.5) | 13 (27.1) | 0.288 |
| *Antiarrhythmic drugs n (%)* | 5 (3.8) | 0 (0.0) | 0.582 | 11 (4.1) | 4 (8.3) | 0.179 |
| *Lipid lowering treatment n (%)* | 16 (12.0) | 0 (0.0) | 0.164 | 61 (22.8) | 10 (20.8) | 0.467 |
| *ACE inhibitors n (%)* | 11 (8.3) | 2 (13.3) | 0.389 | 29 (10.8) | 9 (18.8) | 0.098 |
| *Calcium antagonists n (%)* | 7 (5.3) | 1 (6.7) | 0.584 | 28 (10.4) | 3 (6.3) | 0.273 |
| *Diuretics n (%)* | 4 (3.0) | 2 (13.3 | 0.113 | 24 (9.0) | 5 (10.4) | 0.459 |
| *ARB n (%)* | 14 (10.5) | 1 (6.7) | 0.534 | 24 (9.0) | 4 (8.3) | 0.575 |
| *Digoxin n (%)* | 0 (0.0) | 0 (0.0) |  | 1 (0.4) | 0.0 | 0.848 |
| *PPI n (%)* | 16 (12.0) | 2 (13.3) | 0.572 | 69 (25.7) | 14 (29.2) | 0.369 |
| *Immunosuppressors n (%)* | 9 (6.8) | 3 (20.0) | 0.106 | 35 (13.1) | 8 (16.7) | 0.318 |
| *Corticosteroids n (%)* | 8 (6.0) | 2 (13.3) | 0.268 | 31 (11.6) | 11 (22.9) | 0.034 |
| *Thyroid disease drugs n (%)* | 18 (13.5) | 2 (13.3) | 0.671 | 35 (13.1) | 5 (10.4) | 0.408 |

*ACE: angiotensin converting enzyme, ALT: alanine aminotransferase, ARB: angiotensin receptor blockers, AST: aspartate transaminase, BMI: body mass index, PPI: proton pump inhibitor*

**Supplementary Table 2. Clinical characteristics according to thrombocytopenia degree.**

|  | Mild Thrombocytopenia (N = 36) | Moderate-Severe Thrombocytopenia (N: 27) | P value |
| --- | --- | --- | --- |
| Clinical features | | | |
| *Age (years)* | 57.94±15.83 | 56.50±14.04 | 0.712 |
| *Women n (%)* | 21 (58.3) | 13 (48.1) | 0.422 |
| *BMI mean* | 26.26±4.77 | 24.45±3.12 | 0.091 |
| *Cancer history n (%)* | 4 (11.1) | 2 (7.4) | 0.620 |
| *Diabetes mellitus n (%)* | 2 (5.6) | 3 (11.1) | 0.419 |
| *Hypertension n (%)* | 15 (41.7) | 10 (37.0) | 0.710 |
| *Cerebral Vascular Disease n (%)* | 8 (22.2) | 2 (7.4) | 0.111 |
| *Ischemic Heart Disease n (%)* | 0 (0.0) | 3 (11.1) | 0.040 |
| *Heart Failure (%)* | 1 (2.8) | 1 (3.7) | 0.836 |
| *Peripheral Artery Disease n (%)* | 0 (0.0) | 0 (0.0) | - |
| *Chronic Lung Disease n (%)* | 2 (5.6) | 1 (3.7) | 0.733 |
| *Liver Cirrhosis n (%)* | 0 (0.0) | 1 (3.7) | 0.244 |
| *Chronic Kidney Disease n (%)* | 1 (2.8) | 3 (11.1) | 0.179 |
| *Hypothyroidism n (%)* | 4 (11.1) | 3 (11.1) | 1.000 |
| *Hyperthyroidism n (%)* | 1 (2.8) | 1 (3.7) | 0.836 |
| *Anaemia n (%)* | 2 (5.6) | 3 (11.1) | 0.419 |
| *Haemolytic Anaemia n (%)* | 2 (5.6) | 3 (11.1) | 0.419 |
| *Smoking Habit n (%)* | 9 (25.0) | 3 (11.1) | 0.165 |
| *Livedo reticularis n (%)* | 2 (5.6) | 2 (7.4) | 0.765 |
| Frailty risk elements | | | |
| *Cognitive impairment n (%)* | 5 (13.9) | 2 (7.4) | 0.418 |
| *Bed rest syndrome n (%)* | 1 (2.8) | 0 (0.0) | 0.383 |
| *Falling predisposition n (%)* | 0 (0.0) | 1 (3.7) | 0.244 |
| *Former major bleeding n (%)* | 1 (2.8) | 1 (3.7) | 0.836 |
| *Alcohol consumption n (%)* | 1 (2.8) | 2 (7.4) | 0.393 |
| Previous thrombotic event | | | |
| *Deep Vein Thrombosis n (%)* | 17 (47.2) | 14 (51.9) | 0.716 |
| *Pulmonary Embolism n (%)* | 7 (19.4) | 6 (22.2) | 0.787 |
| *Superficial Vein Thrombosis n(%)* | 3 (8.3) | 0 (0.0) | 0.124 |
| Laboratory indexes | | | |
| *Haemoglobin (g/dl)* | 12.97±1.90 | 13.23±1.89 | 0.589 |
| *Leukocytes (cells/mm^3^)* | 5.92±1.91 | 6.26±2.09 | 0.555 |
| *Creatinine (mg/dl)* | 1.06±0.56 | 0.96±0.34 | 0.418 |
| *AST (U/L)* | 25.0  [17.0-31.0] | 27.0  [19.0-36.5] | 0.510 |
| *ALT (U/L)* | 23.0  [14.5-32.0] | 22.5  [14.8-31.0] | 0.989 |
| *Triple Positivity Pattern n (%)* | 13 (36.1) | 14 (51.9) | 0.212 |
| Therapy | | | |
| *Antiplatelet n (%)* | 10 (27.8) | 9 (33.3) | 0.634 |
| *Antiarrhythmic drugs n (%)* | 3 (8.3) | 1 (3.7) | 0.456 |
| *Lipid lowering treatment n (%)* | 7 (19.4) | 3 (11.1) | 0.370 |
| *ACE inhibitors n (%)* | 4 (11.1) | 7 (25.9) | 0.125 |
| *Calcium antagonists n (%)* | 1 (2.8) | 3 (11.1) | 0.179 |
| *Diuretics n (%)* | 6 (16.7) | 1 (3.7) | 0.105 |
| *ARB n (%)* | 3 (8.3) | 2 (7.4) | 0.893 |
| *Digoxin n (%)* | 0 (0.0) | 0 (0.0) |  |
| *PPI n (%)* | 10 (27.8) | 6 (22.2) | 0.616 |
| *Immunosuppressors n (%)* | 4 (11.1) | 7 (25.9) | 0.125 |
| *Corticosteroids n (%)* | 4 (11.1) | 9 (33.3) | 0.031 |
| *Thyroid disease drugs n (%)* | 5 (13.9) | 2 (7.4) | 0.418 |

*ACE: angiotensin converting enzyme, ALT: alanine aminotransferase, ARB: angiotensin receptor blockers, AST: aspartate transaminase, BMI: body mass index, PPI: proton pump inhibitor*

**Supplementary Table 3. Multivariable stepwise logistic regression of clinical factors associated with moderate-to severe thrombocytopenia.**

|  | Odds Ratio (95% confidence interval) | P value |
| --- | --- | --- |
| Triple positivity pattern | 5.44 (2.27-13.04) | <0.001 |
| Myocardial Infarction | 6.52 (1.59-26.77) | 0.009 |

**Supplementary Figure 1. Antiphospholipid antibodies in antiphospholipid syndrome patients and carriers, first determination. Panel A: anticardiolipin IgM and IgG, Panel B: anti β-glycoprotein I IgM and IgG, Panel C: lupus anticoagulants positivity.**

**A**

*aCL IgM: anticardiolipin IgM; aCL IgG: anticardiolipin IgG*

**B**

*aBGPI IgM: anti-β-glycoprotein I IgM, aBGPI IgG: anti-β-glycoprotein I IgG*

**C**

*LAC + (%): proportions of patients with lupus anticoagulant positive.*

**Supplementary Figure 2. Antiphospholipid antibodies in antiphospholipid syndrome patients and carriers, second determination. Panel A: anticardiolipin IgM and IgG, Panel B: anti β-glycoprotein I IgM and IgG, Panel C: lupus anticoagulants positivity.**

**A**

*aCL IgM: anticardiolipin IgM; aCL IgG: anticardiolipin IgG*

**B**

*aBGPI IgM: anti-β-glycoprotein I IgM, aBGPI IgG: anti-β-glycoprotein I IgG*

**C**

*LAC + (%): proportions of patients with lupus anticoagulant positive.*
